# Changes in levels of enzymes and osmotic adjustment compounds in key species and their relevance to vegetation succession in abandoned croplands of a semiarid sandy region

**DOI:** 10.1002/ece3.6067

**Published:** 2020-02-03

**Authors:** Liu Yang, Liming Lai, Jihua Zhou, Qiaoyan Li, Sangui Yi, Qinglin Sun, Yuanrun Zheng

**Affiliations:** ^1^ Key Laboratory of Resource Plants West China Subalpine Botanical Garden Institute of Botany Chinese Academy of Sciences Beijing China; ^2^ University of Chinese Academy of Sciences Beijing China

**Keywords:** community succession, comprehensive drought evaluation, drought resistance, Ordos Plateau

## Abstract

Reclamation of cropland from grassland is regarded as a main reason for grassland degradation; understanding succession from abandoned cropland to grassland is thus crucial for vegetation restoration in arid and semiarid areas. Soil becomes dry when cropland is reverted to grassland, and enzyme and osmotic adjustment compounds may help plants to adapt to a drying environment. Croplands that were abandoned in various years on the Ordos Plateau in China, were selected for the analysis of the dynamics of enzymes and osmotic adjustment compounds in plant species during vegetation succession. With increasing number of years since abandonment, levels of superoxide dismutase increased in *Stipa bungeana*, first decreased and then increased in *Lespedeza davurica* and *Artemisia frigida*, and fluctuated in *Heteropappus altaicus*. Levels of peroxidase and catalase in the four species fluctuated; levels of proline, soluble sugar, and soluble protein either decreased or first increased and then generally decreased. According to a drought resistance index, the drought resistance of the four species was ranked in descending order as follows: *S. bungeana* > *A. frigida* > *H. altaicus* > *L. davurica*. The drought resistance ability of the different species was closely linked with vegetation succession from communities dominated by annual and biennial species (with main accompanying species of *L. davurica* and *H. altaicus*) to communities dominated by perennial species (*S. bungeana* and *A. frigida*) when soil became dry owing to increasing evapotranspiration after cropland abandonment. The restoration of *S. bungeana* steppe after cropland abandonment on the Ordos Plateau is recommended both as high‐quality forage and for environmental sustainability.

## Graphical abstract

Enzyme and osmotic adjustment compounds of different species were used to build *D* index to evaluate drought resistance ability of key species. The drought resistance of the four species was ranked in descending order as follows: *Stipa bungeana* > *Artemisia frigida* > *Heteropappus altaicus* > *Lespedeza davurica*. The restoration of *S. bungeana* steppe after cropland abandonment on the Ordos Plateau is recommended.

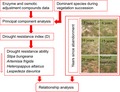

## 1 INTRODUCTION

Approximately 10%–20% of arid and semiarid areas throughout the world are changing dramatically as a result of desertification and grasslands degradation (Reynolds et al., 2007). There are two main causes of desertification: global factors, such as global climate change and rising global CO_2_ concentrations, and local factors, such as chronic high levels of herbivory, overgrazing, grassland reclamation, and combinations of these factors (Belayneh & Tessema, 2017; Reynolds et al., 2007). To effectively restore degraded grassland resulting from reclamation, vegetation succession after cropland abandonment has been a key focus of scholars in the 21st century (Kawada, Wu, & Nakamura, 2015; Ward, Hoffman, & Collocott, 2014). In arid and semiarid areas, abandoned croplands usually revert to three community types: weeds, perennial grass with shrubs or native grass (Cai et al., 2018; Nie, Yuan, Kepner, Erickson, & Jackson, 2012; Reynolds et al., 2007; Wheeler, Archer, Asner, & Mcmurtry, 2007); therefore, interactions between shrub and grass species are crucial for driving vegetation succession of abandoned croplands (Howard, Eldridge, & Soliveres, 2012; Reynolds et al., 2007).

Bottom‐up controls (resources such as water and nitrogen) and top‐down controls (such as fire and herbivory) are commonly used to explain shrub and grass interactions (Cipriotti, Aguiar, Wiegand, & Paruelo, 2014). Niche separation, one of the bottom‐up hypotheses, assumes that shrubs have deeper roots and use deeper soil water compared with grasses; therefore, deeper infiltration of soil water favors shrubs in arid areas (Cipriotti et al., 2014; O'Connor, Puttick, & Hoffman, 2014). The two‐layer hypothesis states that the selective use of deep soil water increases the persistence of shrub species in arid areas, and the resource pool hypothesis proposes that shallow soil water benefits the growth of all plants and that deep soil water benefits only shrub species (O'Connor et al., 2014). Although most hypotheses suggest that water use strategies of grass species and woody species play key roles in the vegetation succession process in degraded grasslands, there are few studies that directly observe the long‐term water use strategies of multiple woody and grass species.

Aerobic metabolism can provide energy for plant growth and development but also generates reactive oxygen species (ROS). Normally, there is a dynamic balance between the intracellular generation and removal of ROS. When plants are under drought stress, the balance of ROS is disrupted, and cells are injured by excessive ROS (Fang & Xiong, 2015). To protect cells from the damage caused by excessive ROS, plants have developed a series of defense mechanisms that make use of antioxidant enzymes such as superoxide dismutase (SOD), peroxidase (POD), and catalase (CAT) (Gill & Tuteja, 2010).

Superoxide dismutase is considered the first line of defense against ROS (Sayfzadeh et al., 2011). SOD is a major scavenger of superoxide anion radicals, and it can catalyze into hydrogen peroxide and oxygen (Scandalios, 1993). POD plays a key role in preventing oxidative damage; it can decrease H_2_O_2_ accumulation and maintain cell membrane integrity (Chakhchar et al., 2015) because of its low substrate affinity (Weng, Cui, Liu, Zhang, & Shan, 2015). CAT is another effective enzyme that can break down H_2_O_2_ (Chakhchar et al., 2015; Weng et al., 2015); it degrades H_2_O_2_ and prevents the subsequent degradation of fatty acids by peroxisomes (Gill & Tuteja, 2010). Malondialdehyde (MDA) is the product of membrane lipid peroxidation and is an indicator of oxidative damage (Sedaghat, Sarvestani, Emam, & Bidgoli, 2017). MDA is also one of the main compounds in drought‐damaged plants (Xu, Zhou, Wang, Han, & Li, 2008).

Another significant drought resistance strategy is osmotic regulation (Deligoz & Gur, 2015; Fang & Xiong, 2015). When exposed to drought, plants accumulate osmotic solutes (soluble sugar [SS] and soluble protein [SP]) to increase the liquid concentration of cells, reduce the osmotic potential to facilitate water absorption, maintain cell turgor, and ensure cell growth and metabolism (Morgan, 1984). When soil moisture levels change markedly, changes in osmotic adjustment compounds that help plants adapt to such changes may provide clues to understanding shrub and grass interactions.

The Ordos Plateau is a typical Chinese ecological transition zone that may be classified as an agriculture and animal husbandry ecotone, and because of the influence of overgrazing, land reclamation, and climatic change, the zonal vegetation (*Stipa bungeana*) has been severely degraded, and a semishrub species (*Artemisia ordosica*) has become dominant (Cai et al., 2018). There are some reports related to grass and shrub interactions, such as grass or shrub dominance are at alternative stable states in the processes of vegetation succession (Peng et al., 2013), climate and native grassland vegetation significantly influenced the community structures of grasslands with high abundance of shrub (Chen et al., 2015), cover of shrub patches significantly influenced the community structures, evapotranspiration and soil organic carbon of grassland ecosystems (Li et al., 2019; Wang et al., 2018; Zhou et al., 2018). Grassland degradation on the Ordos Plateau might have originated during the Yuan Dynasty (600–700 years ago), and during this long history, the plant community composition and soil physical and chemical properties have changed to varying degrees; therefore, the Ordos Plateau may be the ideal location for studying the interactions between shrubs and grass (Cai et al., 2018). Field observations have indicated that after abandonment, croplands reclaimed by semishrub dominated grasslands could be restored to native *S. bungeana* grasslands through long‐term vegetation succession from bare land, to annual, biennial, semishrub, and then perennial species. During this successional process, at the first stage, when annual grasses dominate, the soil water content is relatively high, and at the last stage, when perennial grasses are dominant and community cover is greater, the soil water content is relatively low; therefore, in this semiarid environment, the dominant species in different stages will have different abilities to resist drought (Cai et al., 2018), and enzymes and osmotic adjustment compounds may play an important role in the abilities of species to maintain drought resistance. Plants resist drought through four basic mechanisms: drought avoidance, drought tolerance (DT), drought escape, and drought recovery (Fang & Xiong, 2015). DT refers to the ability of plants to maintain a normal level of physiological and biochemical activities while under water stress by adjusting a series of metabolic pathways to reduce damage from drought stress (Passioura, 1998). To cope with the damaging effects of drought, plants have developed several DT‐associated strategies, which mainly include systems of antioxidant enzymes (such as SOD, POD, and CAT) and osmotic responses (such as changes in the levels of proline and SS) (Fang & Xiong, 2015; Ge et al., 2014). However, the role of these enzymes and osmotic adjustment compounds in plant species in this type of succession is still unclear.

We hypothesized that *S. bungeana* in later successional stages has higher drought tolerance than does *Heteropappus altaicus* in early successional stages. To test this hypothesis, we chose croplands on the Ordos Plateau of Inner Mongolia that had been abandoned at different times and measured the changes in enzyme activity, MDA, and osmotic adjustment compounds in the main species, including grass and semishrub species. The objectives of this study are as follows: (a) to understand how the levels of enzyme activity, MDA, and osmotic adjustment compounds of main species changed during vegetation succession in abandoned cropland and (b) to understand their roles in vegetation succession in abandoned cropland. Answering these questions will improve our understanding of water use strategies of grass and shrub species and help restore degraded grasslands.

## 2 MATERIALS AND METHODS

### 2.1 Study area

The Ordos Plateau (37°35′24″–39°29′37.6″N, 106°42′40″–111°27′20″E) is located on the western margin of the temperate monsoon region, elevation 850–1,600 m, and it measures approximately 400 km from east to west and 340 km from north to south. The research site is in Ejin Horo Town, in the middle of Ejin Horo Banner, in abandoned cropland at 39°24–39°26′N and 109°50′–109°52′E on the Ordos Plateau. At the research site, annual average temperature is 6.2°C, the annual maximum temperature is 35.9°C (in July), and the annual minimum temperature is −20.3°C (in January). Annual precipitation is 357.3 mm. Annual sunshine duration is 2,828.7 hr. Before the croplands were abandoned, the zonal vegetation was a warm‐temperate *S. bungeana* steppe. Corn was planted in croplands; manure and urea were used, but no pesticide was used. After abandonment, the abandoned croplands were succeeded by three community types: weeds, perennial *S. bungeana* with *Artemisia frigida*, and *S. bungeana* (Cai et al., 2018). Grazing was halted for vegetation restoration; grazing only happened occasionally and with very low grazing density.

### 2.2 Experimental design

Field experiments were conducted in July 2017. We selected typical abandoned corn cropland on hard ridge (3, 6, 10, 15, and 20 years since abandonment, based on the information provided by the landowners), and the control was uncultivated natural vegetation near abandoned corn croplands. To ensure similar environments for all sampling plots, all plots were set within 2 km in similar terrain and landform. The species composition of the natural vegetation was similar to the vegetation before the corn croplands were established. Three separate replicates were set up for each age of abandoned cropland and natural vegetation with the same cropping history and the same time since abandonment. In each replicate of each age of abandoned cropland and natural vegetation, three leaf‐sampling plots (20 m × 20 m) were established, and the topmost fully developed functional leaves (upper portion of stems) were collected from over 10 plants chosen randomly in the morning of 1 day. Leaf samples were collected from the four species that were found in all sample plots: *S. bungeana*, *Lespedeza davurica*, *A. frigida,* and *H. altaicus*. All species were deciduous. Leaf samples were immediately placed in a foam box with dry ice, brought to the laboratory, and kept in a −80°C ultralow temperature freezer.

Superoxide dismutase, POD, CAT, MDA, proline (Pro), SS, and SP concentrations were analyzed separately with SOD, POD, CAT, MDA, Pro, SS, and SP assay kits (Comin Biotechnology Co., Ltd.; Pan et al., 2016). Leaf samples that had been maintained at −80°C were ground into a powder with liquid nitrogen. Using a sodium phosphate (Na_2_HPO_4_/NaH_2_PO_4_) buffer, SOD, POD, CAT, MDA, Pro, and SP were extracted by homogenizing on ice (0.1 g leaf tissue for each SOD, POD, CAT, MDA, Pro, and SP assay with 1 ml buffer). To isolate the supernatants for the SOD, POD, CAT, and MDA assays, the homogenates were centrifuged at 8,000 *g* at 4°C for 10 min. For the Pro assay, the homogenates were shaken in a boiling water bath (90°C) for 10 min, cooled and then centrifuged at 1,000 *g* at 25°C for 10 min. For the SP assay, the homogenates were centrifuged at 10,000 *g* at 4°C for 10 min. For the SS assay, the leaf samples that had been maintained at −80°C were ground into a powder in liquid nitrogen, then 0.1 g leaf tissue was homogenized with 1 ml distilled water and maintained in a boiling water bath for 10 min. After cooling, the mixture was centrifuged at 8,000 *g* at 25°C for 10 min to produce the supernatant.

In each 20 m × 20 m leaf‐sampling plot, quadrats covering 5 m × 5 m for semishrub species and 1 m × 1 m for grass species were established. To ensure accurate measurements of biomass, leaves were not collected in these quadrats for analyses of enzyme activity, MDA, or osmotic adjustment compounds. To obtain the aboveground biomass, the aboveground parts of every species were harvested separately, taken to the laboratory and dried to a constant weight at a temperature of 80°C (Cai et al., 2018).

In each 20 m × 20 m leaf sample plot, intact soil cores were collected randomly using a cutting ring (volume of 100 cm^3^) from five soil depths (0–5, 5–10, 10–20, 20–30, and 30–40 cm) after removing any rocks and litter. After collecting the soil samples, we immediately measured the fresh weight (FW), and then, the samples were taken to the laboratory and oven‐dried at 105°C to a constant weight to measure the dry weight (DW). Soil water content (SWC) was calculated as

$$\text{SWC}\left(\%\right)=\frac{\left(\text{FW}-\text{DW}\right)}{\text{FW}}.$$

### 2.3 Statistical analysis

To evaluate the drought resistance ability of a species, principal component analysis (PCA) was used to develop an index (Wold, Esbensen, & Geladi, 1987). Averaged data from three replicates for each of the seven parameters (SOD, POD, CAT, MDA, SS, Pro, and SP) related to the drought resistance of *S. bungeana*, *L. davurica*, *A. frigida,* and *H. altaicus* in different abandoned cropland and natural vegetation were converted into seven principal components for analysis, and then, the results of the PCA were used to build a drought resistance index.

The weight (*W_i_*) and the comprehensive evaluation index (*D*) of each plant were estimated using the following equations (Wang, Nie, Lu, Cui, & Wang, 2015):

(1) $$W_{i}=P_{i}/\sum_{i=1}^{n}P_{i}\;i=1,2,3,\;\ldots ,\;n,$$

(2) $$D=\sum_{i=1}^{n}\left[U_{i}\times w_{i}\right]\;i=1,2,3,\;\ldots ,\;n,$$

where *P_i_* is the contribution rate of principal component *i*, *U_i_* is the subordinative function of principal component *i*, and *D* is the drought resistance index of the species. The higher the *D* value, the higher the drought resistance.

Within each of three replicates, samples were collected from three subplots; data from three subplots were averaged as one datum point for each replicate. A statistical analysis was performed by two‐way ANOVA. If significant differences were found, Duncan's test was used to determine mean differences between treatments (*p* < .05) (Kabacoff, 2015). The relationships among SOD, POD, CAT, MDA, Pro, SS, and SP were examined using Pearson's correlation analysis. All statistical analyses, including the test for homogeneity of variance, were performed using SPSS Statistics 17.0 (SPSS Inc.).

## 3 RESULTS

### 3.1 Changes in the levels of enzymes, MDA, and osmotic adjustment compounds in different species and their correlations

In general, *F* values were significant for the responses of SOD, POD, CAT, MDA, Pro, SS, and SP to species, years since abandonment and their interactions (Table 1).

**Table 1:** Results of a two‐way ANOVA with species and years since abandonment (three replicates). *F* values are shown

Parameter	Effect		
	Years since abandonment (A)	Species (S)	A × S
SOD	108.587***	2,623.444***	81.225***
POD	67.942***	7,516.849***	72.246***
CAT	3,035.603***	62,608.521***	1,963.105***
MDA	18.437***	49.492***	32.655***
Pro	39,794.482***	238,488.837***	10,859.645***
SS	347.859***	2,274.656***	931.659***
SP	109.309***	812.314***	442.791***
*df*	5	3	15

Note

Enzymes, malondialdehyde, and osmotic adjustment compounds were analyzed for four species (*Stipa bungeana*, *Lespedeza davurica*, *Artemisia frigida,* and *Heteropappus altaicus*) in abandoned croplands at five different durations of abandonment (3, 6, 10, 15, and 20 years) and in natural vegetation. The residual degrees of freedom are 48.

Abbreviations: CAT, catalase; MDA, malondialdehyde; POD, peroxidase, Pro, proline; SOD, superoxide dismutase; SP, soluble protein; SS, soluble sugar.

Significance levels:

*** *p* < .001.

**Figure 1:**
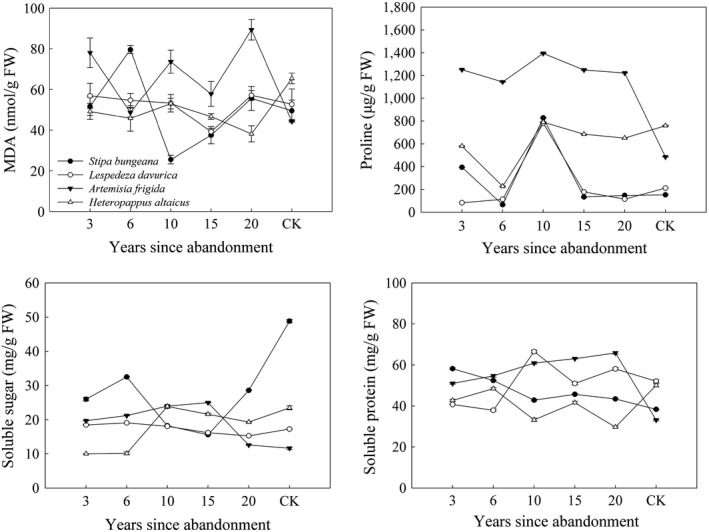
Changes in MDA, Pro, SS, and SP (mean ± *SE*). Each symbol in lines represents the mean of three replicates; results of multivariable comparison are shown in Table A3. Abbreviations are shown in Table 1

Different species showed different patterns with increasing number of years since abandonment. In *S. bungeana*, SOD increased, Pro increased and then decreased, SS decreased and then increased, and SP showed a decreasing trend. In *L. davurica*, SOD decreased and then increased, while Pro, SS, and SP increased and then decreased or slowly decreased. In *A. frigida*, SOD decreased and then increased, while Pro, SS, and SP increased and then decreased. In *H. altaicus*, SOD fluctuated, while Pro, SS, and SP increased or decreased and then increased. POD, CAT, and MDA fluctuated in these four species (Table 2, Table A3, and Figure 1).

**Table 2:** Changes in SOD, POD, and CAT (mean ± *SE*, U/g FW)

Parameter	Years since abandonment	Species			
		*Stipa bungeana*	*Lespedeza davurica*	*Artemisia frigida*	*Heteropappus altaicus*
SOD	3	513.53 ± 7.24 e	333.10 ± 3.32 a	214.88 ± 3.59 c	337.08 ± 3.37 b
	6	561.16 ± 3.45 d	267.56 ± 1.18 c	268.86 ± 1.00 b	310.99 ± 3.42 c
	10	454.06 ± 13.54 f	181.57 ± 2.30 d	206.97 ± 2.80 c	285.39 ± 6.40 d
	15	617.30 ± 23.97 c	179.01 ± 2.19 d	222.31 ± 0.52 c	337.96 ± 6.40 b
	20	755.23 ± 13.01 a	294.28 ± 7.36 b	162.38 ± 4.02 d	378.23 ± 5.15 a
	CK	674.03 ± 9.49 b	281.05 ± 6.78 bc	353.10 ± 11.68 a	251.29 ± 2.48 c
POD	3	603.81 ± 17.81 b	0.43 ± 0.02 b	16.89 ± 0.54 b	75.97 ± 1.79 a
	6	629.72 ± 21.96 b	0.25 ± 0.01 d	16.47 ± 0.57 b	51.40 ± 1.08 d
	10	863.15 ± 11.85 a	0.46 ± 0.02 a	13.54 ± 0.50 c	61.22 ± 1.20 c
	15	596.31 ± 12.52 b	0.29 ± 0.01 c	16.52 ± 0.41 b	47.44 ± 0.79 d
	20	425.41 ± 15.86 c	0.29 ± 0.01 c	10.84 ± 0.30 d	73.33 ± 1.42 a
	CK	583.01 ± 17.94 b	0.30 ± 0.01 c	18.60 ± 0.70 a	66.74 ± 1.46 b
CAT	3	438.16 ± 0.77 a	67.10 ± 2.39 a	90.53 ± 1.22 c	102.27 ± 0.72 c
	6	293.11 ± 0.61 d	33.52 ± 0.99 c	82.35 ± 0.29 d	111.15 ± 0.11 b
	10	384.01 ± 0.43 b	29.80 ± 1.35 cd	140.46 ± 1.69 a	95.87 ± 1.39 d
	15	164.95 ± 0.60 f	39.68 ± 0.99 b	83.70 ± 0.63 b	70.75 ± 0.35 e
	20	317.79 ± 1.43 c	42.68 ± 0.81 b	131.57 ± 0.82 b	69.38 ± 0.67 c
	CK	221.85 ± 2.00 e	28.07 ± 1.05 d	92.82 ± 0.81 c	114.30 ± 1.18 a

Note

Data with different lowercase letters are significantly different from each other with different years of abandonment for same species at *p* < .05 (Duncan's test). One unit of SOD activity (U) was defined as the amount of enzyme that caused a 50% decrease in the SOD‐inhibited nitroblue tetrazolium (NBT) reduction. One unit of POD activity (U) was defined as the amount of enzyme required to increase the absorbance at 470 nm by 0.01 min^−1^ ml^−1^. One unit of CAT activity (U) was defined as the amount of enzyme catalyzing the decomposition of 1 nmol of H_2_O_2_/min. Abbreviations are shown in Table 1.

Significant correlations existed among all or some parameters of SOD, POD, CAT, MDA, Pro, SS, and SP for all four species, and the pairwise correlations among the seven parameters could be positive or negative depending on the species (Table 3).

**Table 3:** Pearson correlation coefficients for enzymes, malondialdehyde, and osmotic adjustment compounds in four species (*n* = 18)

Species	Parameter	POD	CAT	MDA	Pro	SS	SP
*Stipa bungeana*	SOD	−0.851**	−0.521*	0.289	−0.709**	0.430	−0.438
	POD		0.288	−0.475*	0.793**	−0.341	−0.016
	CAT			−0.021	0.624**	−0.180	0.534*
	MDA				−0.686**	0.466	0.420
	Pro					−0.436	−0.037
	SS						−0.286
*Lespedeza davurica*	SOD	−0.094	0.574*	0.570*	−0.651**	0.131	−0.537*
	POD		0.350	0.153	0.632**	0.268	0.396
	CAT			0.184	−0.464	0.072	−0.443
	MDA				−0.027	0.272	−0.063
	Pro					0.168	0.756**
	SS						−0.488*
*Artemisia frigida*	SOD	0.722**	−0.529*	−0.846*	−0.873**	−0.302	−0.899**
	POD		−0.787**	−0.757**	−0.560*	0.085	−0.729**
	CAT			0.656**	0.363	−0.126	0.428
	MDA				0.621**	−0.056	0.597**
	Pro					0.687**	0.886**
	SS						0.490*
*Heteropappus altaicus*	SOD	0.169	−0.749**	−0.877**	−0.210	−0.351	−0.562*
	POD		0.069	0.004	0.293	−0.117	−0.302
	CAT			0.649**	−0.307	−0.307	0.717**
	MDA				0.378	0.385	0.546*
	Pro					0.828**	−0.401
	SS						−0.311

Note

Abbreviations are shown in Table 1.

Significance levels:

* *p* < .05;

** *p* < .01.

### 3.2 Change in levels of enzymes, MDA, and osmotic adjustment compounds in different plant functional types

**Figure 2:**
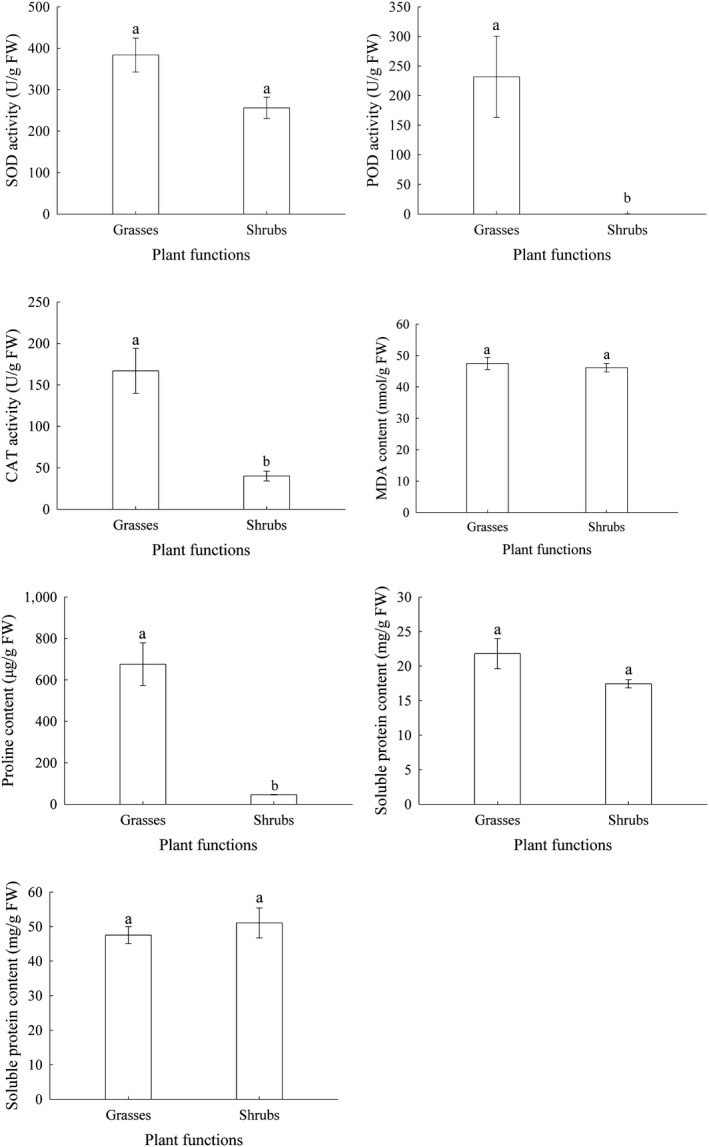
Changes in MDA, Pro, SS, and SP (mean ± *SE*) in two plant functions (grass and semishrub). Each bar represents the mean of three replicates; bars with different lowercase letters are significantly different from each other with different plant functions at *p* < .05 (Duncan's test). Abbreviations are shown in Table 1

Levels of POD, CAT, and Pro were significantly higher for grasses than they were for semishrubs (*p* < .05), while there were no significant differences between grasses and semishrubs in SOD, MDA, SS, or SP. The average levels of POD, CAT, and Pro for grasses were 689.13, 4.16, and 2.73 times higher than those of semishrubs, respectively (Figure 2).

### 3.3 Change in aboveground biomass

With increasing number of years since abandonment, aboveground biomass increased for *S. bungeana*, increased and then decreased for *L. davurica* and *A. frigida*, and declined for *H. altaicus* (Table 4).

**Table 4:** Aboveground biomass (mean ± *SE*) of four species on the Ordos Plateau in abandoned croplands since different abandonment years (g/m^2^)

Years since abandonment	*Stipa bungeana*	*Lespedeza davurica*	*Artemisia frigida*	*Heteropappus altaicus*
3	18.7 ± 2.8	0.2 ± 0.04	3.6 ± 0.6	7.2 ± 2.6
6	23.8 ± 4.1	5.8 ± 2.1	5.1 ± 2.8	4.4 ± 2.3
10	52.9 ± 4.4	3.9 ± 1.7	0.5 ± 0.4	1.5 ± 1.3
15	133.7 ± 30.5	1.1 ± 1.0	4.1 ± 1.2	2.5 ± 0.9
20	157.0 ± 27.3	1.1 ± 0.9	3.2 ± 2.0	2.5 ± 1.2
CK	161.4 ± 7.1	0.3 ± 0.1	0.1	0.8

### 3.4 Comprehensive drought resistance index (*D*)

The contribution rate was over 90% for the first two principal components, which contained almost all information from the measured parameters. The eigenvalue of the first principal component was 4.582, its contribution rate was 65.5%, and the corresponding characteristic vectors of SOD, POD, CAT, and SS were large. The eigenvalue of the second principal component was 1.809, its contribution rate was 25.8%, and the corresponding characteristic vectors of SP and MDA were large (Table 5, Table A1).

**Table 5:** Results of principal component analysis

Principal component	Eigenvalue	Contribution rate (%)	Cumulative contribution rate (%)
1	4.582	65.460	65.460
2	1.809	25.838	91.298
3	0.609	8.702	100.000
4	1.333E‐16	1.904E‐15	100.000
5	−1.229E‐16	−1.755E‐15	100.000
6	−1.608E‐16	−2.297E‐15	100.000
7	−2.825E‐16	−4.036E‐15	100.000

Based on the standardized characteristic vector, the linear combinatorial equations of the first two principal components and the seven parameters were obtained:

(3) $$C\left(1\right)=0.461X_{1}+0.449X_{2}+0.420X_{3}+0.425X_{4}-0.291X_{5}-0.220X_{6}-0.309X_{7},$$

(4) $$C\left(2\right)=0.117X_{1}+0.205X_{2}+0.312X_{3}+0.308X_{4}+0.425X_{5}+0.512X_{6}+0.557X_{7},$$

where *X*_1_, *X*_2_, … *X*_7_ were SOD, POD, CAT, MDA, Pro, SS, and SP, respectively.

Based on the contribution rates of the first two principal components and Equation 1, *W_i_* was calculated, and based on the values of *W_i_*, *C*(*i*), *U_i_* and Equation 2, *D* was calculated.

The *D* values indicated that among these four species, the drought resistance decreased as follows: *S. bungeana* (0.907) > *A. frigida* (0.283) > *H. altaicus* (0.247) > *L. davurica* (0.182) (Table A2).

### 3.5 Coefficients of variation of different parameters of the four species

For the seven parameters measured, the coefficients of variation (CVs), the ratios of standard deviation to the mean, varied depending on the species. Among CVs of different parameters, the value for SOD was large for *L. davurica* and *A. frigida*, the values for POD and CAT were large for *S. bungeana* and *L. davurica*, the value for Pro was large for *S. bungeana* and *L. davurica*, the values for SS and MDA were large for *S. bungeana* and *H. altaicus*, and the value for SP was small for all four species (Table 6).

**Table 6:** Coefficients of variation for four species

Parameter	*Stipa bungeana* (%)	*Lespedeza davurica* (%)	*Artemisia frigida* (%)	*Heteropappus altaicus* (%)
SOD	17.59	23.13	26.18	13.40
POD	21.81	24.16	17.66	17.66
CAT	31.22	33.95	23.26	19.67
MDA	19.45	8.00	16.39	10.17
Pro	94.09	101.03	27.02	31.19
SS	39.43	7.78	28.12	33.49
SP	14.49	19.66	20.43	18.66

Abbreviations are shown in Table 1

## 4 DISCUSSION

### 4.1 Effects of enzyme activity, MDA, and osmotic adjustment compounds

Studies have shown that when plants suffer from drought stress, the antioxidant enzyme activity increases, but when the degree of stress exceeds a certain threshold, the antioxidant enzyme activity decreases (Chakhchar et al., 2015; Ge et al., 2014). Excess active oxygen species caused by water stress can cause membrane lipid peroxidation. MDA can be an indicator of oxidative damage (Sedaghat et al., 2017). Many studies have shown that when plants are subjected to water stress, MDA levels increase (Jia, Sun, Li, Li, & Chen, 2015), and low MDA is associated with drought resistance, which can improve plant growth (Bacelar et al., 2007). In addition, water stress can increase the accumulation of osmotic regulators (Jia et al., 2015), but there have also been several studies that have found no increase in levels of osmotic regulators (Deligoz & Gur, 2015).

In our study, antioxidant enzyme activity varied among durations since abandonment and among species, indicating that the species may encounter different degrees of drought stress and that soil water content generally declines after abandonment because different species may consume different amounts of soil water (Table A4). The roots of the perennial grass in this study were mainly located in the 0–10 cm soil layer, and soil bulk density decreased with increasing time since abandonment, which was unfavorable for the use of water for deep‐rooted species. However, during the last stages, as the *S. bungeana* community developed, soil water in the shallow layer may decrease because of high community coverage. For *S. bungeana*, SOD was negatively correlated with POD and CAT, indicating SOD, POD, and CAT may show different responses to soil water. For *L. davurica*, SOD and CAT activities were positively correlated, which showed that these two enzymes cooperated to remove excess ROS. However, the CVs of the three enzymes were all large, which indicated that *L. davurica* was sensitive to water stress and showed poor drought resistance. For *A. frigida*, the POD and CAT activities fluctuated with small CVs, indicating a high level of drought resistance. For *H. altaicus*, the activity of antioxidant enzymes decreased in the early stage and increased later, and the CVs of the three enzymes were small, indicating a certain level of drought resistance. The MDA of the four species either fluctuated with very small CVs and showed a clear correlation with antioxidant enzyme activity, indicating that MDA was linked to the increases in antioxidant enzyme activity and antioxidant ability, the elimination of ROS, and the decreased membrane lipid peroxidation damage (Esfandiari, Shekari, Shekari, & Esfandiari, 2007; Ge et al., 2014). Levels of Pro and SP showed a clear correlation, and the CVs of Pro and SS were large, indicating *S. bungeana* was sensitive to water stress. Pro may play a different role in drought resistance mechanisms; it can act as a scavenger of free radical species to protect cells from oxidative damage (Girija et al., 2002) or as a reserve for plant assimilation of nitrogen and carbon after stress (Silveira et al., 2003). Decreases in Pro levels in our study may be due to its role in the transition between drought resistance mechanisms (Silva, Ferreira‐Silva, Viégas, & Silveira, 2010).

### 4.2 Drought resistance of the four species and implications

The four species differed in their resistance to drought. The various parameters showed that the four species had different mechanisms of drought resistance. *S. bungeana* is a perennial grass species with a root system mainly distributed in the 0–10‐cm soil layer. *A. frigida* is a perennial grass species or occasional small semishrub species with a deep root system that reached the 1‐m soil layer and was mainly distributed in the 30‐cm soil layer. *L. davurica* is a semishrub species with a deep root system that reached the 1.3‐m soil layer and was mainly distributed in the 30‐cm soil layer. *H. altaicus* is a perennial grass species with a deep root system that reached the 1‐m soil layer and was mainly distributed in the 0–20‐cm soil layer. Antioxidant enzyme activity was higher in *S. bungeana* than in the other species, which indicated that *S. bungeana* had strong drought resistance. The Pro level was higher in *A. frigida* than in other species, which indicated that *A. frigida* was more tolerant to water stress and had higher drought resistance (Siddiqui et al., 2016). The drought resistance index (*D*) indicated that the drought resistance ranks of the four species in decreasing order were *S. bungeana* > *A. frigida* > *H. altaicus* > *L. davurica*, which was basically consistent with the field species distribution data in plant communities. On the Ordos Plateau, after abandonment of cropland, annual grass species occurred during the first stage, then perennial semishrub species and perennial grass species occurred in the intermediate stages, and perennial grass with some semishrub species occurred during the final stage (Cai et al., 2018). *A. frigida*, *H. altaicus*, and *L. davurica* were the accompanying species in a typical *S. bungeana* steppe in a semiarid area on the Loess Plateau (Cai et al., 2018). At the initial stage after cropland abandonment, community coverage and transpiration were low, and soil water was relatively high, especially in the deep layer in sandy soil. The soil gradually improved from the first stage to the last stage after cropland abandonment as fine clay particles accumulated in the surface soil layer, and seepage of rain to deep soil decreased. Therefore, the soil water content increased in the shallow soil layers and decreased in the deep soil layers. This change is beneficial for shallow‐rooted species but not for deep‐rooted species, as suggested by the two‐layer hypothesis (Cipriotti et al., 2014). This pattern was also evident in this study; the aboveground biomass of *S. bungeana* increased, that of *H. altaicus* decreased and that of *L. davurica* was relatively stable, with a decreasing trend for that of *A. frigida* (Table A4). The results of this study clearly indicate why the four species differ in drought resistance abilities, mainly because of their long‐term adaptation to different soil water environments, and our results were also consistent with the two‐layer hypothesis (Cipriotti et al., 2014).

Based on the trends of soil changes and drought resistance for the studied species, recommended practices for restoring *S. bungeana* steppe for high forage value and favorable environmental effects include reducing soil disturbance, accelerating the accumulation of fine clay particles, improving the soil structure, and increasing the soil water in the shallow layer.

## 5 CONCLUSIONS

Based on analyses of enzymes, MDA, osmotic adjustment compounds, and drought resistance, we found that the four studied species (*S. bungeana*, *L. davurica*, *A. frigida,* and *H. altaicus*) have efficient adaptive mechanisms that enhance their ability to resist drought by upregulating their antioxidant and osmotic adjustment systems in response to drought stress. A comprehensive evaluation showed that the drought resistance of the four species could be ranked in descending order as follows: *S. bungeana* > *A. frigida* > *H. altaicus* > *L. davurica*. After cropland abandonment, *S. bungeana* in later successional stages had higher levels of enzyme and osmotic adjustment compounds and therefore had greater drought resistance ability compared with other species in early successional stages during vegetation succession as soil became drier. Antioxidant enzymes, MDA, and osmotic adjustment compounds could be good indicators for understanding vegetation succession.

## CONFLICT OF INTEREST

None declared.

## AUTHOR CONTRIBUTIONS

Liu Yang and Yuanrun Zheng designed the work and the experiment. Liu Yang, Liming Lai, Jihua Zhou, Qiaoyan Li, Sangui Yi, and Qinglin Sun participated in field experiment. Liu Yang analyzed the statistics and wrote the draft and Yuanrun Zheng revised the draft carefully, and all remaining authors contributed to the review of the manuscript.

## Data Availability

Leaf enzyme activities, MDA, osmotic adjustment compounds, and aboveground biomass data have been deposited in the Dryad Digital Repository. https://doi.org/10.5061/dryad.931zcrjg7

## APPENDIX 1

**Table A1:** Component matrix of principal component analysis

Parameter	Principal component	
	1	2
SOD	0.987	0.158
POD	0.961	0.276
CAT	0.900	0.419
SS	0.910	0.414
Pro	−0.622	0.571
SP	−0.471	0.688
MDA	−0.661	0.749

Note

Abbreviations are shown in Table 1

**Table A2:** Values of the component score *C_i_*, subordinate function *U_i_*, and comprehensive evaluation *D* of the four species

Species	*C*_1_	*C*_2_	*U*_1_	*U*_2_	*D*	Order
*Stipa bungeana*	3.035	0.629	1.000	0.671	0.907	1
*Lespedeza davurica*	−0.932	−0.899	0.197	0.143	0.182	4
*Artemisia frigida*	−1.904	1.582	0.000	1.000	0.283	2
*Heteropappus altaicus*	−0.199	−1.313	0.345	0.000	0.247	3
Weighting			0.717	0.283		

**Table A3:** Results of multivariable comparison of MDA, Pro, SS, and SP

Parameter	Species	Years of abandonment					
		3	6	10	15	20	CK
MDA	*Stipa bungeana*	b	a	d	c	b	b
	*Lespedeza davurica*	a	a	a	b	a	a
	*Artemisia frigida*	b	d	b	c	a	d
	*Heteropappus altaicus*	bc	f	a	c	d	b
Pro	*Stipa bungeana*	b	f	a	e	d	c
	*Lespedeza davurica*	e	d	a	c	d	b
	*Artemisia frigida*	b	d	a	b	c	e
	*Heteropappus altaicus*	e	f	a	c	d	b
SS	*Stipa bungeana*	d	b	e	f	c	a
	*Lespedeza davurica*	b	a	c	e	f	d
	*Artemisia frigida*	d	c	b	a	e	f
	*Heteropappus altaicus*	d	d	a	b	c	a
SP	*Stipa bungeana*	a	b	d	c	d	e
	*Lespedeza davurica*	d	e	a	c	b	c
	*Artemisia frigida*	e	d	c	b	a	f
	*Heteropappus altaicus*	b	a	c	b	d	a

Note

Different lowercase letters indicate values of MDA, Pro, SS, and SP are significantly different from each other with different years of abandonment for same species at *p* < .05 (Duncan's test). Abbreviations are shown in Table 1.

**Table A4:** Soil water content (Mean ± *SE*) in abandoned croplands since different abandonment years and natural vegetation (%) on the Ordos Plateau

Soil depth (cm)	Years of cropland abandonment					
	3	6	10	15	20	CK
0–5	3.8 ± 0.7	3.1 ± 0.6	2.8 ± 0.7	2.1 ± 0.5	4.6 ± 1.1	4.6 ± 0.8
5–10	5.1 ± 0.6	4.5 ± 0.5	4.4 ± 0.5	3.2 ± 0.5	4.5 ± 0.7	5.4 ± 0.5
10–20	5.6 ± 0.5	5.7 ± 0.5	6.1 ± 0.5	4.6 ± 0.7	4.6 ± 0.4	8.1 ± 0.9
20–30	5.5 ± 0.4	7.4 ± 0.9	6.6 ± 0.6	3.5 ± 0.5	4.3 ± 0.5	8.2 ± 1.5
30–40	5.5 ± 0.3	6.7 ± 0.7	5.6 ± 0.5	2.1 ± 0.4	3.4 ± 0.3	7.1 ± 1.2
